# Spontaneous rupture of adrenal pheochromocytoma in a patient with Von Recklinghausen's disease

**DOI:** 10.4103/0972-5229.56056

**Published:** 2009

**Authors:** Ramin Azhough, Ali Reza Barband, Negar Motayagheni, Mitra Niafar, Hojjat Pourfathi

**Affiliations:** **From:** Departments of General Surgery, Endocrinology and Anesthesiology, Tabriz University (Medical Sciences)

**Keywords:** Adrenal tumor, pheochromocytoma, von Recklinghausen's disease, retroperitoneal hematoma

## Abstract

Spontaneous rupture of an adrenal pheochromocytoma is extremely rare and can be lethal because of dramatic changes in the circulation. We describe a 35-year-old Iranian female with previously diagnosed von Recklinghausen's disease who suffered spontaneous rupture of an adrenal pheochromocytoma, misdiagnosed as renal colic followed by an extensive retroperitoneal hematoma, irreversible hemodynamic shock, and death.

## Introduction

Neurofibromatosis Type 1 (NF-1), or von Recklinghausen's disease, is one of the neurocutaneous syndromes also known as the phakomatoses. Although there may be as many as eight different clinical subgroups, Type 1 and Type 2 account for 99% of the cases.[1] Pheochromocytoma is a rare tumor of chromafin cells most commonly arising from the adrenal medulla and often in association with the familial multiple endocrine neoplasia syndromes (MEN, Type 2A and 2B), neurofibromatosis, von Hippel-Lindau disease, cerebellar hemangioblastoma, Sturge-Webers syndrome, and tuberous sclerosis.[2,3] We report a case of a patient with previously diagnosed von Recklinghausen's disease who presented with left upper quadrant pain, misdiagnosed as renal colic, followed by massive retroperitoneal hematoma and a fatal outcome due to a spontaneous rupture of an adrenal pheochromocytoma.

## Case Report

**Figure 1:**
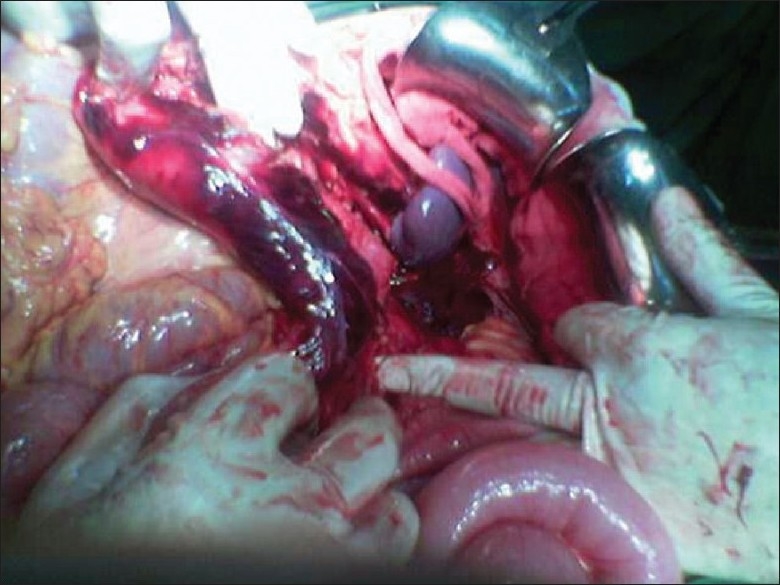
Hemorrhagic adrenal mass on operation

A 35-year-old female was admitted to our hospital following a syncopal episode followed by pain in the left upper quadrant, radiating to the left flank accompanied by nausea and vomiting. She had a history of von Recklinghausen's disease (café au lait spots and numerous cutaneous nodules visible over her skin). Upon presentation, her blood pressure was 160/90 without significant oscillation and her pulse rate was 100/min. She had no previous history of hypertension. An abdominal examination had no abnormal finding except for a mild tenderness on percussion of the left hypochondriac region. In the emergency department, she underwent an abdominal ultrasound examination which showed mild hydronephrosis of the left ureter. There was no stone or mass in the kidneys. She was considered to have renal colic, which was conservatively treated with an antiemetic and analgesic, and she was discharged after pain relief and normalization of blood pressure. The patient was admitted to the hospital again 20 hours after the initial discharge, in a state of shock. Her blood pressure was undetectable, her heart rate was 140, and mucosal surfaces were dry and pale. A bedside abdomimal ultrasound examination revealed copious amount of free intra-abdominal fluid. After initial resuscitation, she immediately underwent an exploratory laparotomy. Following incision, a significant amount of free blood was found in the abdominal cavity. The retroperitonium contained an extensive hematoma. On exploration we found a hemorrhagic left adrenal tumor measured 1 × 1 cm, somewhat adhering to the kidney [Figure 1]. A left adrenalectomy was performed immediately and the patient was transferred to the surgical intensive care unit but despite good intensive care and comprehensive advanced resuscitation, the patient expired due to irreversible hemorrhagic shock. Histopathological examination of the tumor demonstrated a hemorrhagic pheochromocytoma.

## Discussion

Families with von Recklinghausen's disease, which is now called neurofibromatosis, have been divided clinically into those without (neurofibromatosis 1) and with (neurofibromatosis 2) acoustic neuromas. Clinical diagnosis of von Recklinghausen's disease is based on the presence of two or more signs of six or more café au lait spots, two or more neurofibromas or one plexiform neurofibroma, axillary or inguinal freckling, optic glioma, two or more Lisch nodules (hamartomas of the iris), osseous lesions (sphenoid dysplasia, pseudarthrosis or scoliosis) or one sign and a first degree affected relative. Pheochromocytoma occurs in a small but defined number of patients with von Recklinghausen's disease (in 0.1 to 5.7% of patients with von Recklinghausen's disease), and can be associated with significant morbidity and mortality if not detected. Patients experience a lifetime of morbidity and increased risk of mortality, depending on the extent of the disease. An adrenal mass can be incidentally discovered in any patient and must be evaluated in those with von Recklinghausen's disease to exclude pheochromocytoma. Patients with von Recklinghausen's disease also have adrenal ganglioneuromas, which can be mistaken clinically for nonfunctional pheochromocytomas. Medullary hyperplasia has been reported with von Recklinghausen's disease but the clinical significance of this finding is not yet known.[4] Most patients with spontaneous rupture of pheochromocytoma are admitted to the hospital due to acute abdominal pain. In majority of cases, the bleeding occurs in the retroperitonium; however, some may bleed both in the retroperitonium and peritoneal cavity,[5] such as in the present case. The exact mechanism of a pheochromocytoma rupture is unknown, but a high intracapsular pressure may tear the capsule and also cause necrosis of the tumor. The high pressure may be caused by rapid tumor growth or intratumoral hemorrhage. Elevation of blood pressure is probably associated with vasoconstriction in the tumor and subsequent necrosis, causing massive release of catecholamine into circulation.[6] Our pathological findings of hemosiderin deposition and coagulation necrosis supported this assumption.

Pheochromocytoma with hemorrhagic shock, especially in a clinically silent case of von Recklinghausen's disease is an extremely rare condition; however, it should always be taken into consideration in order to avoid a delay in diagnosis and the possibility of progression to death. Since pheochromocytoma is not common, evaluation of all patients with von Recklinghausen's disease with abdominal pain might be reasonable.
